# High Protein Diet Induces Oxidative Stress in Rat Cerebral Cortex and Hypothalamus

**DOI:** 10.3390/ijms20071547

**Published:** 2019-03-28

**Authors:** Ewa Żebrowska, Mateusz Maciejczyk, Małgorzata Żendzian-Piotrowska, Anna Zalewska, Adrian Chabowski

**Affiliations:** 1 Department of Physiology, Medical University of Bialystok, 15-089 Bialystok, Poland; mat.maciejczyk@gmail.com (M.M.); adrian@umb.edu.pl (A.C.); 2 Department of Hygiene and Epidemiology, Medical University of Bialystok, 15-089 Bialystok, Poland; mzpiotrowska@gmail.com; 3 Department of Restorative Dentistry, Medical University of Bialystok, 15-089 Bialystok, Poland; azalewska426@gmail.com

**Keywords:** high protein diet, oxidative stress, oxidative damage, cerebral cortex, hypothalamus

## Abstract

This is the first study to analyze the impact of high protein diet (HPD) on antioxidant defense, redox status, as well as oxidative damage on both a local and systemic level. Male Wistar rats were divided into two equal groups (*n* = 9): HPD (44% protein) and standard diet (CON; 24.2% protein). After eight weeks, glutathione peroxidase (GPx), glutathione reductase (GR), catalase (CAT), superoxide dismutase-1 (SOD-1), reduced glutathione (GSH), uric acid (UA), total antioxidant (TAC)/oxidant status (TOS) as well as advanced glycation end products (AGE), 4-hydroxynonenal (4-HNE), and malondialdehyde (MDA) were analyzed in the serum/plasma, cerebral cortex, and hypothalamus of HPD and CON rats. HPD resulted in higher UA concentration and activity of GPx and CAT in the hypothalamus, whereas in the cerebral cortex these parameters remained unchanged. A significantly lower GSH content was demonstrated in the plasma and hypothalamus of HPD rats when compared to CON rats. Both brain structures expressed higher content of 4-HNE and MDA, whereas AGE was increased only in the hypothalamus of HPD animals. Despite the enhancement in antioxidant defense in the hypothalamus, this mechanism does not protect the hypothalamus from oxidative damage in rats. Hypothalamus is more susceptible to oxidative stress caused by HPD.

## 1. Introduction

A high protein diet (HPD) exerts many beneficial effects in the condition of overweight, metabolic syndrome, bone health, and cardiovascular risk factors [1,2]. Changes in body composition, weight loss, and a decrease in total and visceral fat content in obese people ingesting high amounts of protein may result from a reduced food intake, increased energy expenditure, and increased fatty acid oxidation, as well as increased thermogenesis [3,4]. However, while short-term use of such diets usually brings positive effects in obese patients, there are only few reports on the positive effects of this diet in non-obese patients. It has been shown that in highly developed countries, protein intake is twice as high as WHO recommendation [5] and undesired metabolic changes occur when the protein intake is 1.6 or more than the recommended values [6]. Therefore, both the physiological and functional consequences of long-term high-protein intake should be investigated.

Recent studies show that potential adverse effects of HPD are mainly associated with modifications in amino acid metabolism as well as in alterations in the acid–base balance caused by increased dietary acid load and may be responsible for impairment in kidney and liver functioning and even increased cancer risk [7]. Furthermore, HPDs rich in branched-chain amino acids (BCAAs) in combination with a high fat diet promote insulin resistance in rats [8]. Increased oxidation of amino acids (resulting from a higher protein intake) may enhance mitochondrial oxygen radical generation and lead to oxidative stress (OS) if the antioxidant defense is disrupted [9]. Oxidative damage caused by excessive protein ingestion was previously observed in kidneys, liver, pancreas, and salivary glands [10,11,12]. However, it is still not known what role OS plays in different brain structure impairment caused by chronic ingestion of high amounts of protein.

Studies concerning the effects of HPD on brain functioning are mainly focused on understanding the satiating effect of protein on the brain regions involved in energy homeostasis (brainstem and hypothalamus) [13]. Some studies have shown the beneficial effects of HPD on rodents’ brains such as preventing cerebral ischemia and reducing apoptosis in the ischemic cortex [14]. However, there are also reports of an adverse effect of a chronic HPD on the brain functioning. HPD is probably responsible for worsening spatial memory deficits in cirrhotic mice [15]. What is more, the ingestion of this type of diet in a mouse model of Alzheimer disease caused a 5% loss in brain weight and neuronal density [16].

The brain is particularly sensitive to damage induced by OS mainly due to its high oxygen consumption, high content of phospholipids and polyunsaturated fatty acids (highly susceptible to oxidants), redox-active metals abundance, and finally, a low activity of antioxidant enzymes [17,18,19,20]. Thus, OS is considered to be a major pathogenic factor in many neurodegenerative disorders including Alzheimer’s and Parkinson’s disease, schizophrenia, and other cognitive impairments [20,21,22,23]. Since HPD may disrupt redox balance in different organs, it may also be responsible for the brain complications induced by an increased dietary protein intake. Therefore, the main goal of our study was to investigate whether HPD induces OS both in the brain and plasma and to compare the response of different brain structures involved in cognition processes (cerebral cortex) and energy homeostasis (hypothalamus).

## 2. Results

### 2.1. Effects of High Protein Diet on Body Weight and Plasma Metabolic Parameters

The average daily food intake was significantly lowered in the HPD (animals fed a high protein diet) group (−23%) when compared to the CON (animals fed a standard diet) group; however, all the studied animals had similar energy intake and final body weight (Table 1). Similarly, glucose homeostasis was not affected by the eight weeks of the HPD administration since both glucose, insulin concentrations, and HOMA-IR (homeostatic model assessment of insulin resistance) were similar to the CON animals. We did not observe any differences in plasma adiponectin and leptin concentration between the studied groups. Estimated protein intake was higher (+117%) in the HPD group when compared to CON. Total protein content in both the cerebral cortex and hypothalamus remained unchanged in the HPD animals when compared to CON animals (Table 1).

### 2.2. Non-Enzymatic and Enzymatic Antioxidants, Total Antioxidant/Oxidant Status, and Oxidative Damage Product Level in Plasma/Serum

To assess the antioxidant barrier, we determined the concentration of non-enzymatic antioxidants (GSH, reduced glutathione; UA, uric acid), activity of antioxidant enzymes (CAT, catalase; GPx, glutathione peroxidase; GR, glutathione reductase; SOD-1, superoxide dismutase-1) as well as redox status (TAC, total antioxidant capacity; TOS, total oxidant status; OSI, oxidative stress index; FRAP, ferric reducing ability of sample). GSH concentration in the plasma of HPD fed rats was significantly lowered (−58%) when compared to the CON animals (Table 2). The activity of GPx and CAT was significantly higher (+45% and +325%, respectively) in the HPD group, whereas SOD-1 activity was lower (−21%) when compared to the CON group. In contrast, GR activity in the serum of the studied animals remained unchanged. UA content was significantly higher (+80%) in the animals fed HPD as compared to the CON. TOS and FRAP were also greater (+104% and +32%, respectively) whereas TAC and OSI remained unchanged in the HPD group when compared to the CON group.

Determination of oxidative stress level was based on assessment of oxidative damage to proteins (AGE, advanced glycation end products) and lipids (4-HNE, 4-hydroxynonenal; MDA, malondialdehyde). All of the estimated oxidative damage products (AGE, 4-HNE, and MDA) were significantly higher (+25%, +268%, and +29%, respectively) in the plasma of the HPD animals (Table 2).

### 2.3. Pro-Oxidant Enzymes and Antioxidants in Cerebral Cortex and Hypothalamus

**Figure 1:**
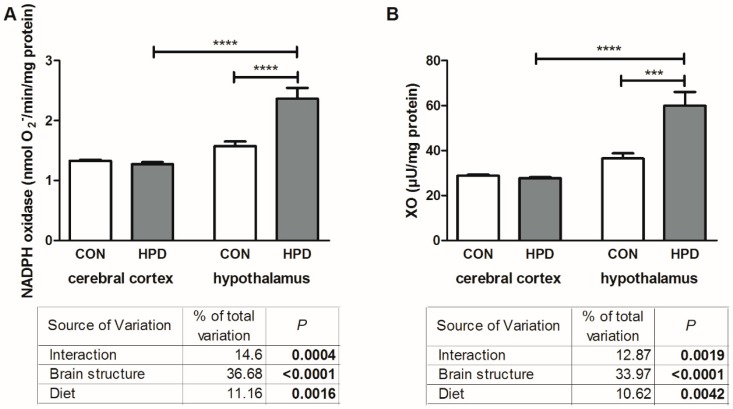
Effect of 8-week HPD on pro-oxidant enzymes (NADPH oxidase (**A**), XO (**B**)) in rat cerebral cortex and hypothalamus. Values are means ± SEMs, *n* = 9. Differences statistically significant at: *** *p* < 0.0005, **** *p* < 0.0001. CON, standard diet; HPD, high protein diet; XO, xanthine oxidase.

The results of two-way ANOVA analysis for pro-oxidant enzymes (NADPH oxidase and XO, xanthine oxidase) showed significant effects for the brain structure, diet, as well as the interaction between the brain structure and diet (Figure 1).

The activity of NADPH oxidase and XO in the hypothalamus was markedly higher in HPD than in CON (+64% and +50%, respectively), whereas in the cerebral cortex they remained unchanged (Figure 1). What is more, we observed a significant difference in the activity of NADPH oxidase and XO between both studied brain structures but only in animals fed HPD (+85% and +116%, respectively in the hypothalamus vs cerebral cortex).

**Figure 2:**
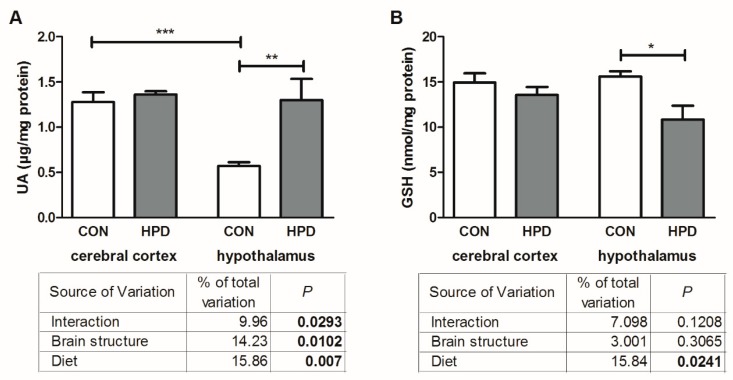
Effect of 8-week HPD on non-enzymatic antioxidants (uric acid (**A**), reduced glutathione (**B**)) in rat cerebral cortex and hypothalamus. Values are means ± SEMs, *n* = 9. Differences statistically significant at: * *p* < 0.05, ** *p* < 0.005, *** *p* < 0.0005. CON, standard diet; GSH, reduced glutathione; HPD, high protein diet; UA, uric acid.

The results of two-way ANOVA analysis for non-enzymatic antioxidant UA showed significant effects for the brain structure, diet, as well as the interaction between the brain structure and diet. In contrast, for GSH content the analysis showed a significant effect only for the diet (Figure 2).

The concentration of UA was significantly higher in the hypothalamus of HPD fed rats in comparison to CON (+128%), whereas in the cerebral cortex no significant differences between the studied groups were observed (Figure 2). On the other hand, the lowered GSH content in the hypothalamus of the HPD rats (−23%) was observed as compared to CON, while the cerebral cortex level of GSH remained unchanged. Furthermore, significant differences in UA content between the cerebral cortex and hypothalamus (−56%) were observed only in the CON group, whereas in the HPD group these parameters were similar in both brain structures.

**Figure 3:**
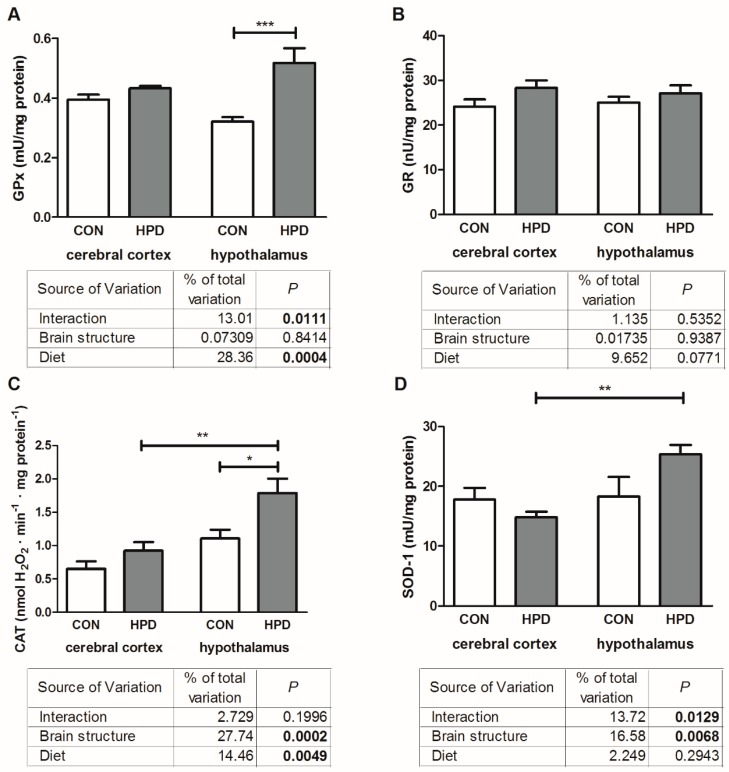
Effect of 8-week HPD on enzymatic antioxidants activity (glutathione peroxidase (**A**), glutathione reductase (**B**), catalase (**C**), superoxide dismutase (**D**)) in rat cerebral cortex and hypothalamus. Values are means ± SEMs, *n* = 9. Differences statistically significant at: * *p* < 0.05, ** *p* < 0.005, *** *p* < 0.0005. CAT, catalase; CON, standard diet; GPx, glutathione peroxidase; GR, glutathione reductase; HPD, high protein diet; SOD-1, superoxide dismutase.

The results of two-way ANOVA analysis for GPx (enzymatic antioxidant) showed significant effects for the diet, as well as the interaction between the brain structure and diet, but not for brain structure alone (Figure 3). For CAT activity, we have shown significant effects for the diet and brain structure, while the interaction between both factors was not significant. Two-way ANOVA analysis for SOD-1 indicated significant effects for the studied brain structures and interaction between the diet and brain structure, whereas diet alone was not the main source of variation. In contrast, no significant effects for GR activity were observed.

The activity of GPx and CAT were significantly higher in the hypothalamus of the HPD animals (+63%, +61%, respectively) when compared to the CON group (Figure 3). In contrast, the activity of SOD-1 was similar in CON and HPD groups. Only the activity of GR was similar for both the cerebral cortex and hypothalamus, independent of the animal feeding. We did not observe any changes in GPx and GR between the studied brain structures, whereas the activity of CAT and SOD-1 was markedly increased in the hypothalamus (+93%, +72%, respectively) of HPD animals.

### 2.4. Total Antioxidant/Oxidant Status in Cerebral Cortex and Hypothalamus

**Figure 4:**
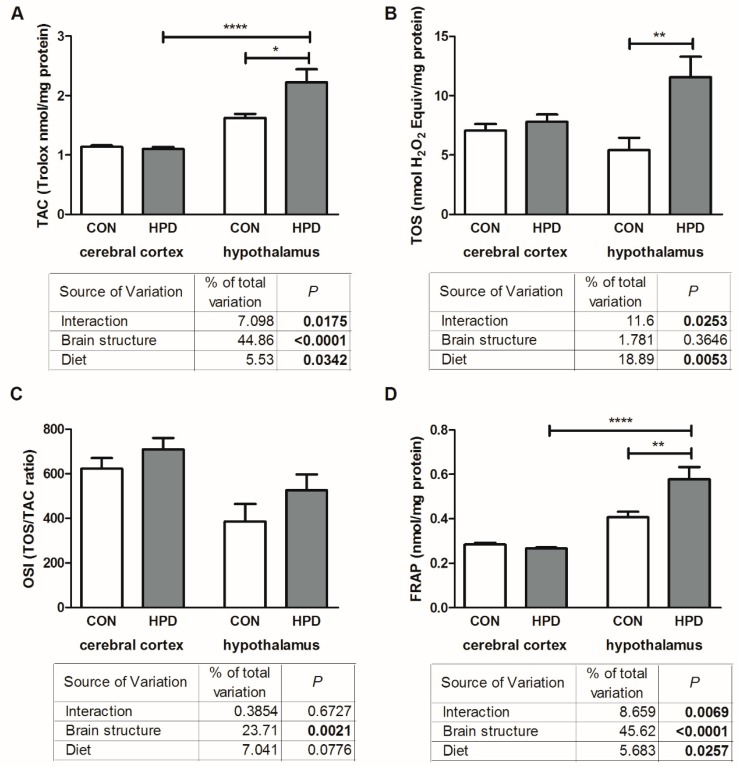
Effect of 8-week HPD on total antioxidant capacity (**A**), total oxidative status (**B**), oxidative stress index (**C**), and ferric reducing ability of sample (**D**), in rat cerebral cortex and hypothalamus. Values are means ± SEMs, *n* = 9. Differences statistically significant at: * *p* < 0.05, ** *p* < 0.005, **** *p* < 0.0001. CON, standard diet; FRAP, ferric reducing ability of sample; HPD, high protein diet; OSI, oxidative stress index; TAC, total antioxidant capacity; TOS, total oxidative status.

The results of two-way ANOVA analysis for TAC and FRAP concentration showed significant effects for the diet, the brain structure, as well as the interaction between them (Figure 4). Two-way ANOVA analysis for TOS indicated significant effects for the diet and interaction between the diet and brain structure, whereas the brain structure alone was not the main source of variation. For OSI we have shown significant effects only for the brain structure, while the diet as well as the interaction between both factors was not significant.

In the hypothalamus of the HPD group, the TOS as well as TAC were significantly higher (+113% and +38%) when compared to the CON group (Figure 4). OSI in the hypothalamus of the HPD animals was similar to CON. There were no significant differences in total antioxidant/oxidant status between the HPD and CON groups in the cerebral cortex. FRAP concentration in the hypothalamus of the HPD animals was significantly higher (+41%) when compared to the CON group (Figure 4). We did not observe any changes in TOS and OSI between the studied brain structures, whereas TAC and FRAP content was markedly increased in the hypothalamus vs. cerebral cortex (+105%, +117%, respectively) but only in HPD animals.

### 2.5. Oxidative Damage Products in Cerebral Cortex and Hypothalamus

**Figure 5:**
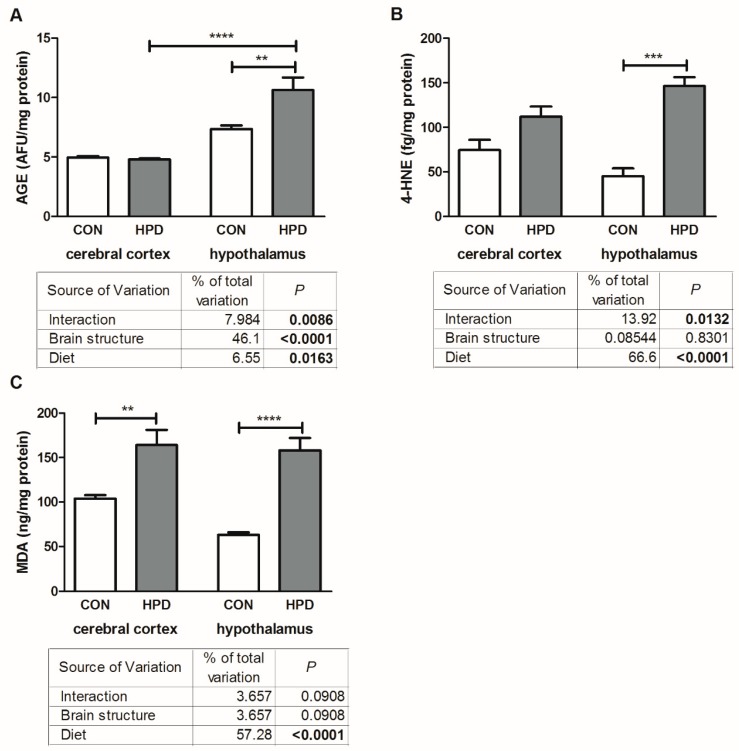
Effect of 8-week HPD on oxidative damage products (advanced glycation end products (**A**), 4-hydroxynonenal (**B**), malondialdehyde (**C**)) in rat cerebral cortex and hypothalamus. Values are means ± SEMs, *n* = 9. Differences statistically significant at: ** *p* < 0.005, *** *p* < 0.0005, **** *p* < 0.0001. 4-HNE, 4-hydroxynonenal; AGE, advanced glycation end products; CON, standard diet; HPD, high protein diet; MDA, malondialdehyde.

The results of two-way ANOVA analysis for AGE content showed significant effects for the brain structure, diet, as well as the interaction between the brain structure and diet (Figure 5). Two-way ANOVA analysis for 4-HNE indicated significant effects for the diet and interaction between the diet and brain structure, but not for brain structure alone. For MDA we have shown a significant effect only for the diet, whereas the brain structure and the interaction between both factors were not significant.

The 4-HNE protein adduct concentration was significantly higher only in the hypothalamus (+226%) of the HPD group when compared to the CON group (Figure 5). MDA concentration was also greater in the hypothalamus (+122%) as well as in the cerebral cortex (+58%) of the HPD animals when compared to CON. Contrasting, AGE, a marker of protein damage, was significantly higher (+44%) only in the hypothalamus of the HPD group when compared to the CON group (Figure 5). What is more, AGE content in the hypothalamus of HPD animals was higher when compared to the cerebral cortex (+122%). We did not observe any differences in 4-HNE and MDA between the studied brain structures.

### 2.6. NFκB Expression and SIRT-1

The results of two-way ANOVA analysis for NFκB (nuclear factor-κB) showed significant effects for the brain structure, as well as the interaction between the brain structure and diet, but not for diet alone (Figure 3). In contrast, no significant effects for SIRT-1 expression were observed.

**Figure 6:**
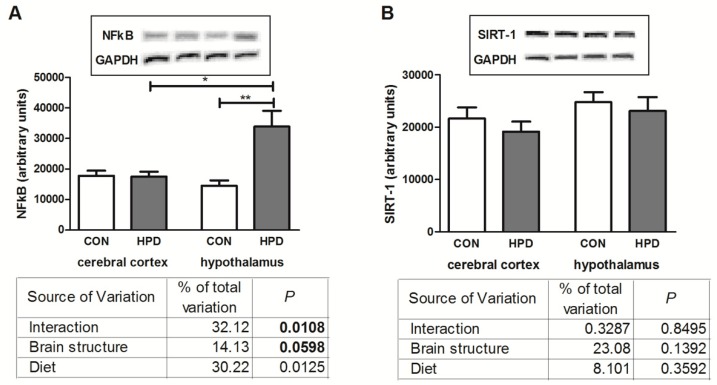
Effect of 8-week HPD on NFκB (**A**) and SIRT-1 (**B**) in rat cerebral cortex and hypothalamus. Values are means ± SEMs. Differences statistically significant at: * *p* < 0.05, ** *p* < 0.005. CON, standard diet; GAPDH, glyceraldehyde 3-phosphate dehydrogenase; HPD, high protein diet; NFκB, nuclear factor κB; SIRT-1, sirtuin 1.

HPD significantly elevated NFκB expression only in the hypothalamus (+112%) (Figure 6). The total expression of SIRT-1 remained unchanged in all examined groups. Additionally, NFκB expression was higher in the hypothalamus of HPD animals when compared to the cerebral cortex (+93%).

## 3. Discussion

As living standards have increased, the amount of protein ingested by people has drastically risen to exceed actual nutritional requirements. Since the brain is particularly prone to oxidative damage and, due to existence of many controversial studies concerning its response to HPD, we have investigated antioxidant status as well as the oxidative damage in different brain structures of rats fed a high protein diet. This is also the first study that has compared redox homeostasis and OS induced by a chronic administration of HPD on both systemic (serum/plasma) and local (cerebral cortex and hypothalamus) levels in healthy animals.

Generally, OS is caused by an imbalance between overproduction of ROS and detoxification of these highly reactive molecules [23]. In our study, we have shown higher activity of the enzymatic antioxidants GPx and CAT, both in the serum and in the hypothalamus, as well as higher levels of UA and TAC in HPD rats. The enhanced antioxidant defense, both on the systemic and the CNS level, may indicate that an HPD leads to overproduction of free radicals, suggesting an adaptive response to protect against cellular OS. The increase in TAC level is particularly important because this parameter reflects the resultant effect of all (enzymatic and non-enzymatic) antioxidative mechanisms [24]. The higher activity of antioxidant enzymes (SOD, CAT) in the brain of HPD fed rats was also demonstrated by Camiletti-Móiron et al. [25]. In our study we also observed a lower content of GSH both in the plasma and hypothalamus, whereas in the cerebral cortex, GSH level was similar to the CON animals. It is well known that GSH is the most important component of the antioxidant brain defense and the only compound that scavenges the hydroxyl radical [17]. This molecule is not just a storage form of cysteine, a neuromodulator/neurotransmitter, but it also regulates apoptosis and neuronal differentiation [17]. It was previously demonstrated that lowered GSH levels leads to mitochondrial damage in the brain [21]. Therefore, the lower GSH content observed in our study could lead to increased oxidative damage despite the fact that that enzymatic antioxidants were markedly increased. Although we did not directly assess the rate of ROS production, the increased intensity of oxidative processes was demonstrated by the elevated TOS in the hypothalamus of HPD fed rats. However, we did not observe any significant differences in the OSI, which may indicate that the brain is trying to balance ROS overproduction (↑TOS) via antioxidative mechanisms (↑TAC).

OS is known to accelerate generation of advanced glycation end products (AGE) [26] and AGE interactions with their receptors (RAGE) elicits OS and oxidative damage [27]. It was demonstrated that AGE may increase the activity of NADPH oxidase and, thus, elevate free radical generation, which results in ROS-induced apoptosis. Additionally, it is well known that NADPH oxidase enhances production of proinflammatory cytokines and therefore higher activity of NADPH oxidase may suggest not only increased production of ROS but also inflammatory response in the hypothalamus of the HPD rats. Indeed, in our study we have shown an enhanced expression of NFκB, which is responsible for activation of proinflammatory and free radical signaling in the brain [28]. However, further examination is needed to confirm HPD effects on neuroinflammation (including immunohistochemical studies).

Increased protein intake leads to enhanced amino acid oxidation to maintain amino acid homeostasis of the organism, as proteins cannot be stored. The reoxidation of reducing equivalents derived from amino acid oxidation may increase free radical generation during electron flow along the mitochondrial respiratory chain [10,11]. In mice digestive systems, an increase in ROS generation was observed as a consequence of HPD [10]. Additionally, it was evidenced that the deleterious effect of HPD on kidney is connected with excessive dietary amounts of AGE in obese and diabetic patients [29]. The HPD animals in our study also expressed a higher level of AGE in the hypothalamus and plasma as compared to CON.

Lipid peroxidation, another important marker of OS occurrence, is responsible for many degenerative changes of brain cell membranes [20]. As the brain contains a large amount of polyunsaturated fatty acids (PUFAs) it is extremely exposed to oxidants’ attack [30]. Free radicals and products of lipid peroxidation may destroy the spatial membrane arrangement and impair membrane enzyme activity, e.g., Na^+^/K^+^ ATPase necessary to maintain the functional activity of nerve cells [31]. Moreover, high MDA and 4-HNE content may damage DNA and protein (by forming various adducts with these molecules) and, therefore, has mutagenic and carcinogenic potential [32]. Deleterious effects of lipid peroxidation products (mainly 4-HNE) on brain neurodegeneration is associated with an increase in blood–brain barrier (BBB) permeability (mainly by alteration of tight junction protein expression) [33]. Similarly, in our study we have observed higher 4-HNE and MDA content in the plasma, cerebral cortex, and hypothalamus of the HPD animals (Figure 5, Table 2). However, having compared both brain structures, the highest content of lipid peroxidation markers was observed in the hypothalamus, which suggests that the hypothalamus is more prone to oxidative damage caused by a chronic HPD, especially considering that AGE content in the cerebral cortex remained unchanged. Recently, it was evidenced that HPD may induce BBB dysfunction in mice [34]. This effect was more pronounced when casein was the protein source (probably because of a high content of homocysteine, derived from methionine, which impairs BBB permeability), while soy seemed to attenuate negative HPD’s effect on BBB integrity [34]. Enhanced lipid peroxidation and protein oxidation in rat brains was also previously reported in HPD fed rats (45% of proteins) [25]. On the other hand, there are some reports showing that HPD (33%, and even 60% of protein) as well as low and a normal protein diet did not induce oxidative damage in mice plasma [35]. Furthermore, it was even suggested that diets rich in protein (27–33%) could even prevent OS-induced toxicity and oxidative damage [36]. In contrast to our findings, Soulsby et al. [37] demonstrated that HPD (with soy as a protein source) prevented MDA from accumulating in the brain of rats subjected to simulated weightlessness. In our study, lipid peroxidation was also enhanced in the plasma of HPD animals. Petzke et al. [9] reported that even a chronically administrated diet containing 60% protein did not increase the reactive carbonyl residues in plasma proteins of adult rats. Interestingly, symptoms of OS were observed only in the first week of HPD. These findings are supported by other studies that confirmed that rats can adapt to HPD within two weeks [38,39]. Despite the OS manifestations observed in our study, we did not notice any adverse effects of HPD on metabolic homeostasis (glucose metabolism, peripheral insulin resistance) as well as rats’ body weight.

It is evidenced that the brain does not respond to OS uniformly [17]. Our results showed that the cerebral cortex and hypothalamus express different responses to HPD in terms of ROS generation/inactivation. This may be attributed to differences in energy metabolism in these brain regions as the main source of ROS generation in the brain is the mitochondrial oxidative chain [19]. It was proven by Villa et al. [40] that in senescent rats the cerebral cortex energy metabolism is less affected, whereas in the hypothalamus enhancement in oxidative metabolism was observed. These findings are consistent with our study, suggesting that the increased OS in the hypothalamus may result from increased oxidative metabolism. Although our study did not assess behavioral changes under the influence of HPD, it is very likely that increased OS can lead to anxiety and depressive-like behavior, as observed in the conditions of high fat or high carbohydrate intake [41]. It is suggested that OS may be the common denominator of these abnormalities. However, the presented hypothesis requires further research and observations.

## 4. Materials and Methods

The study was performed in accordance with the guidelines of the Committee for Ethical Use of Animals at the Medical University of Bialystok, Poland (protocol number 89/2015, 2015/109, 9/06/2015).

Eighteen male Wistar 6-week old rats (67–72 g), were kept under controlled conditions (20 °C ± 2, 12 h light/12 h dark cycle) having constant eye contact with each other. During the acclimation period (7 days) the animals were fed with a commercial rodent chow (24% of energy from protein, 13.5% from fat, and 62.5% carbohydrates; energy value 0.012 mJ/g; Agropol Motycz, Poland). Then the animals were randomly divided into two dietary groups: CON—fed a standard diet (as described above) and experimental—an HPD group (44% energy from protein, 14% from fat, and 33% carbohydrates; energy value 0.0158 mJ/g; Research Diets Inc., D03012801). The animals had unrestricted access to water and food during the acclimation period and the 8 weeks of feeding experiment.

The rats were weighed before the experiment and their body weight as well as food intake were monitored weekly. After 8 weeks (following an overnight fasting) rats were anesthetized by intraperitoneal application of sodium pentobarbital (80 mg/kg body weight). Subsequently, the whole blood was collected from the abdominal aorta into the glass tubes (to obtain serum) and heparinized tubes (to obtain plasma) and centrifuged (3000× *g*, 4 °C, 10 min). After centrifuging the blood, BHT antioxidant was added to the supernatant (10 µL 0.5M BHT in acetonitrile per 1 ml sample) [42]. At the same time the whole cerebral cortex and hypothalamus were excised and immediately freeze-clamped with aluminum tongs precooled in liquid nitrogen. All the samples were stored at −80 °C until biochemical determinations.

Directly before the determinations the plasma and brain tissues were slowly thawed at 4 °C. The cerebral cortex and hypothalamus were homogenized (Omni TH, Omni International, Kennesaw, GA, USA) in ice cold PBS (1:15) and sonicated (20 s, three times, 1800 J/sample; ultrasonic cell disrupter, UP 400S, Hielscher, Teltow, Germany). Then, the homogenates were centrifuged for 20 min at 5000× *g* (MPW Med Instruments, Warsaw, Poland) and supernatants were used for biochemical assays. All the above-mentioned steps were conducted at 4 °C. The brain tissues were treated with protease inhibitor (1 tablet/10 mL PBS; Complete Mini Roche, France) and the antioxidant (100 µL 0.5 M BHT in acetonitrile per 10 mL PBS) [20].

### 4.1. Plasma Insulin, Glucose, Adiponectin, and Leptin Concentrations

Insulin concentration was measured in the plasma with a commercially available ELISA kit according to the manufacturer’s instructions (Abbott, Lake Bluff, IL, USA). The fasting blood glucose concentration was measured with a glucose meter (Accu-Chek Bayer, Germany). The insulin sensitivity was evaluated using the homeostasis model assessment of insulin resistance (HOMA-IR) = fasting insulin (U/mL) × fasting glucose (mM)/22.5 [43].

The concentration of plasma adiponectin and leptin was determined by ELISA method using ready-made kits (Rat Total Adiponectin/Acrp30 Quantikine ELISA Kit, R&D System; Mouse/Rat Leptin Quantikine ELISA Kit, R&D System), according to the manufacturer’s instructions.

### 4.2. Pro-Oxidant Enzymes and Antioxidants in Cerebral Cortex and Hypothalamus

NADPH oxidase and XO activities were analyzed immediately after sample collection. NADPH oxidase activity was measured by luminescence assay using lucigenin as a luminophore [44]. One unit of NADPH oxidase activity was defined as the amount of enzyme required to release 1 nmol of superoxide anion per one minute. XO activity was estimated colorimetrically at 290 nm by measuring the increase in uric acid (UA) absorbance [45]. One unit of XO activity was defined as the amount of enzyme required to release 1 μmol of UA per one minute.

Glutathione peroxidase (GPx) activity was analyzed spectrophotometrically based on the reduction of organic peroxides by GPx in the presence of NADPH [46]. Glutathione reductase (GR) activity was estimated spectrophotometrically by measuring the decrease in absorbance of NADPH at 340 nm [47]. Catalase (CAT) activity was determined in triplicate samples by measuring the decomposition rate of hydrogen peroxide (H_2_O_2_) at 240 nm [48]. Cu–Zn superoxide dismutase-1 (SOD-1) activity was estimated spectrophotometrically by measuring the cytosolic activity of SOD by inhibiting the oxidation of adrenaline at 480 nm [49].

Uric acid (UA) concentrations were measured spectrophotometrically using a commercial kit from BioAssay Systems, Harward, CA, USA (QuantiChromTM Uric Acid DIUA-250 kit), as instructed by the manufacturer. Reduced glutathione (GSH) content was analyzed spectrophotometrically by reaction with 5,5′-dithiobis-2-nitrobenzoic acid (Ellman’s method) [50].

All the assays were performed in duplicate samples (except for CAT determination) in the homogenates of brain samples. The activity of enzymatic antioxidants was also estimated in the serum samples, and concentrations of non-enzymatic antioxidants in the plasma samples. The absorbance/fluorescence was measured using an Infinite M200 PRO Multimode Microplate Reader, Tecan. The results were standardized to one mg of total protein. The total protein concentration was estimated by the bicinchoninic acid (BCA) method [51], using commercial kit Thermo Scientific PIERCE BCA Protein Assay (Rockford, IL, USA).

### 4.3. Total Antioxidant/Oxidant Status

Total antioxidant capacity (TAC) was measured spectrophotometrically using 2,2-azinobis-3-ethylbenzothiazoline-6-sulfonic acid radical cation (ABTS*^+^) [52]. Total oxidant status (TOS) was determined spectrophotometrically based on the oxidation of Fe^2+^ to Fe^3+^ in the presence of oxidants contained in the sample [53]. Oxidative stress index (OSI) was calculated using the formula: OSI = TOS/TAC × 100 [26]. Ferric reducing ability of sample (FRAP) was estimated in triplicate samples using 2,4,6-tripyridyl-s-triazine [54].

All the assays were performed in duplicate samples, except for TAC and FRAP determination, in the homogenates of hypothalamus and cerebral cortex as well as in the plasma. The results were standardized to one mg of total protein.

### 4.4. Oxidative Modification Products

The content of advanced glycation end products (AGE) was determined fluorometrically in 96-well microplates by measuring AGE-specific fluorescence at 350/440 nm [42]. The content of 4-hydroxynonneal protein adducts (4-HNE) was determined using the ELISA method (OxiSelectTM HNE Adduct Competitive ELISA Kit, Cell Biolabs Inc. San Diego, CA, USA). Malondialdehyde (MDA) concentration was estimated spectrophotometrically using the thiobarbituric acid reactive substances (TBARS) method with 1,3,3,3 tetraethoxypropane as a standard [55].

All the assays were performed in duplicate samples in the homogenates of hypothalamus and cerebral cortex as well as in the plasma. The results were standardized to one mg of total protein.

### 4.5. Western Blot Analysis

The Western blot procedure was described in detail by Mikłosz et al. [56] Briefly, tissue homogenate containing 30 µg of total protein was subjected to sodium dodecyl sulfate-polyacrylamide gel electrophoresis (SDS-PAGE) and transferred to nitrocellulose membranes (0.75 A for 1 h). Then, membranes were blocked in Tris Buffer Saline Tween 20 (TBST; 20 mM Tris, 150 mM NaCl, 0.1% Tween 20) containing 5% non-fat dry milk (90 min at room temperature). Membranes were incubated overnight with primary antibodies: SIRT1 (Santa Cruz Biotechnology, Santa Cruz, CA, USA) and anti-nuclear factor-κB (NF-κB) (Cell Signaling Technology, Leiden, The Netherlands). Next, SIRT1 and NFκB were detected with antirabbit IgG horseradish peroxidase-conjugated secondary antibody (Santa Cruz Biotechnology). The protein bands were visualized using a chemiluminescence substrate (Thermo Scientific, Waltham, MA, USA) and quantified by densitometry (Bio-Rad Systems). The protein expression was normalized to glyceraldehyde 3-phosphate dehydrogenase (GAPDH, Santa Cruz Biotechnology) expression.

### 4.6. Statistical Analysis

Statistical analysis was performed using the GraphPad Prism 7 for MacOS (GraphPad Software, La Jolla, CA, USA). Specific analyses included two-way ANOVA and the post hoc Tukey test for honestly significant difference (HSD). Student’s t-test was also used. The threshold for statistical significance was *p* < 0.05.

## 5. Conclusions

Our study proves that a chronically ingested HPD (44% protein) may lead to redox imbalance and OS on the brain as well as on systemic levels in healthy non-obese rats. We have shown that despite the high content of antioxidants (mainly UA and TAC) and enhanced activity of enzymatic antioxidants in the hypothalamus, these mechanisms do not protect against oxidative damage. Interestingly, in the cerebral cortex we have also noticed a higher content of oxidative damage markers (mainly attributed to lipid peroxidation), although to a lesser extend when compared to the hypothalamus. What is more, these damages were not accompanied with enhanced antioxidant defense (both enzymatic and non-enzymatic). The observed differences among the studied brain compartments suggest that the hypothalamus is more susceptible to OS caused by HPD. The lowered level of GSH in the hypothalamus (and in the plasma), despite activation of other antioxidants, may be responsible for an increase in oxidative damage occurrence. Bearing in mind that OS is one of the causative factors of many diseases, the findings of the present study are of great significance in the context of an increase in daily ingested protein.

## Figures and Tables

**Table 1:** Effect of 8-week HPD on rats’ body weight, plasma glucose, insulin, adiponectin, leptin, total protein concentration, food, and energy intake.

Parameter	CON	HPD
Body weight (g)	341 ± 2.49	350 ± 7.57
Glucose concentration (mg/dL)	99.8 ± 2.49	101 ± 5.12
Insulin concentration (µU/mL)	4.75 ± 0.02	4.95 ± 0.14
HOMA-IR	3.08 ± 0.17	3.56 ± 0.14
Adiponectin (µg/mL)	23.4 ± 0.57	22.4 ± 0.55
Leptin (ng/mL)	26.4 ± 0.63	24.8 ± 0.61
Food intake (g/day)	21.2 ± 0.84	16.2 ± 0.63 *
Energy intake (mJ/day)	0.28 ± 0.01	0.27 ± 0.03
Protein energy intake (mJ/day)	0.06 ± 0.05	0.13 ± 0.04 *
Cerebral cortex total protein concentration (µg/mL)	2742 ± 83.1	2796 ± 43.4
Hypothalamus total protein concentration (µg/mL)	1710 ± 66.1	1529 ± 53.3

Values are means ± SEMs, *n* = 9. * difference statistically significant at *p* < 0.05. CON, standard diet; HOMA-IR, homeostatic model assessment of insulin resistance; HPD, high protein diet.

**Table 2:** Effect of 8-week HPD on enzymatic and non-enzymatic antioxidants, total antioxidant/oxidant status, and oxidative damage products in rat plasma/serum.

Parameter	CON	HPD
GPx (mU/mg protein)	0.44 ± 0.02	0.64 ± 0.06 *
GR (nU/mg protein)	11.8 ± 0.77	9.55 ± 0.32
CAT (nmol H_2_O_2_·min^−1^·mg protein^−1^)	6.02 ± 0.49	25.6 ± 1.52 *
SOD-1 (mU/mg protein)	54.3 ± 2.02	42.8 ± 0.65 *
UA (µg/mg protein)	2.71 ± 0.27	4.89 ± 0.58 *
GSH (nmol/mg protein)	7.30 ± 0.71	3.06 ± 0.59 *
TAC (Trolox nmol/mg protein)	3.94 ± 0.13	5.14 ± 0.51
TOS (nmol H_2_O_2_ Equiv/mg protein)	13.5 ± 2.87	27.5 ± 2.22 *
OSI (TOS/TAC ratio)	344 ± 78.5	542 ± 38.9
FRAP (nmol/mg protein)	1.14 ± 0.09	1.51 ± 0.09 *
AGE (AFU/mg protein)	3.26 ± 0.21	4.06 ± 0.21 *
4-HNE (fg/mg protein)	279 ± 62.6	1028 ± 85.7 *
MDA (ng/mg protein)	471 ± 28.7	607 ± 44 *

Values are means ± SEMs, *n* = 9. * difference statistically significant at *p* < 0.05. Enzymatic antioxidants were determined in the serum, whereas other markers—the plasma. 4-HNE, 4-hydroxynonenal; AGE, advanced glycation end products; CAT, catalase; CON, standard diet; FRAP, ferric reducing ability of sample; GPx, glutathione peroxidase; GR, glutathione reductase; GSH, reduced glutathione; HPD, high protein diet; MDA, malondialdehyde; OSI, oxidative stress index; SOD-1, superoxide dismutase-1; TAC, total antioxidant capacity; TOS, total oxidant status; UA, uric acid.

## Abbreviations

4-HNE	4-hydroxynonenal
AGE	advanced glycation end products
BBB	blood–brain barrier
BCA	bicinchoninic acid
BCAA	branched-chain amino acids
BHT	butylated hydroxytoluene
CAT	catalase
CNS	central nervous system
FRAP	ferric reducing ability of plasma
GAPDH	glyceraldehyde 3-phosphate dehydrogenase
GPx	glutathione peroxidase
GR	glutathione reductase
GSH	reduced glutathione
HOMA-IR	homeostatic model assessment of β-cell function and insulin resistance
HPD	high protein diet
MDA	malondialdehyde
NADPH	nicotinamide adenine dinucleotide phosphate
NFκB	nuclear factor-κB
OS	oxidative stress
OSI	oxidative stress index
PBS	phosphate buffered saline
PUFAs	polyunsaturated fatty acids
RAGE	receptor for advanced glycation end products
ROS	reactive oxygen species
SDS-PAGE	sodium dodecyl sulfate-polyacrylamide gel electrophoresis
SIRT-1	sirtuin 1
SOD-1	superoxide dismutase-1
TAC	total antioxidant capacity
TBARS	thiobarbituric acid reactive substances
TNF-alpha	tumor necrosis factor alpha
TOS	total oxidant status
UA	uric acid
WHO	World Health Organization
